# Evaluation of Distress and Stress in Cancer Patients in AMIR Oncology Hospital in Shiraz

**Published:** 2014-12-10

**Authors:** A Mansourabadi, M Moogooei, S Nozari

**Affiliations:** 1 Department of Clinical Immunology, Yazd University of Medical Science, Yazd, Iran.; 2 Department of Clinical Parasitological, Ghazvin University of Medical Science, Ghazvin, Iran.

**Keywords:** Distress, Stress, Distress Thermometer, Brief Symptom Inventory, Hospital Anxiety and Depression Scale

## Abstract

**Background:**

Routine screening for distress is internationally recommended as a necessary standard for good cancer care given its high prevalence and negative consequences on quality of life. This study attempts to support validation of Distress Thermometer (DT) in Shiraz, Iran and in the second step to investigate privilege/priority of DT over other referent criterion measures.

**Material and methods:**

In total, 58 outpatients with cancer were recruited from AMIR Oncology hospital in Shiraz, Iran. Each participant completed the DT and a list of 34 possible cancer-related problems (the Problem List), the Hospital Anxiety and Depression Scale (HADS), the 18-item Brief Symptom Inventory (BSI-18), and a short visual analog scale to determine the understandability of the tools.

**Results:**

Characteristic analysis revealed that DT cutoff scores ≥4 and ≥5 had optimal sensitivity and specificity relative to both HADS and BSI-18 cutoff scores for general caseness and more severe psychological distress, respectively. Patients with DT scores ≥4 (cases) were more likely to be women suffering from psychological problems in the past experience stressful events in the 3 years ago and encounter more family, emotional, and physical issue related to cancer or cancer treatment (p=0.02).

**Conclusion:**

Patients indicated that the DT was easier to fill out and to understand than the HADS, but not the BSI-18. The DT was identified as a simple and effective screening instrument for detecting distress in Iranian cancer patients as a first step toward more properly referring those in need to psychosocial intervention.

## Introduction

Several studies have demonstrated the emotional distress symptoms of 30% to 40% of cancer patients in consequence of the disease and the treatment. Many of who meet the criteria for psychiatric diagnosis, such as adjustment, anxiety, and depressive disorders (1). Although cancer impairs the quality of patients and their families’ life, it leads to longer rehabilitation. In the oncology setting, 33% of cancer patients diagnosed with distress are recognized and referred for proper clinical intervention (2). For aforementioned reasons, several guidelines for psychosocial screening have been developed, and distress has been endorsed as the sixth vital sign to be monitored constantly and routinely across the cancer disease trajectory to identify patients in need of clinical attention and intervention (3-5 ). In fact, it has been demonstrated that clinical judgment alone does not work properly a screening tools. In a study by Mitchell et al. 45% of 401 patients with cancer had distress based on Distress Thermometer (DT) (6). However, Nurses (could identify) identified distress hardly using their routine clinical judgment which led them to make false negative errors (sensitivity, 51%) and, to a lesser degree, false-positive errors (specificity, 80%). Even much worrying finding were recently reported from a large sample of 2642 cancer outpatients in follow-up care by Werner et al. who observed a high prevalence of distress among patients ,but very low identification was made by physicians of those presenting clinically significant distress (1 of 10 patients; patient-physician concordance=0.1) (7). In this background, the DT and the Problem List (PL) have been devised by Distress Management Guidelines Panel within National Comprehensive Cancer Network in the United States (8,9) .Over the last 10 years, the DT has become one of the most used tools and has been applied in several contexts (e.g. outpatient clinics, inpatient units, and palliative care) as well as different phases of cancer trajectories. The present findings confirm DT’s validity as a screening instrument. In a multicenter study of 380 cancer outpatients in the United States, Jacobsen et al. demonstrated that a DT cutoff score ≥4 optimized sensitivity and specificity for detection of patients with emotional distress (caseness) compared with psychological questionnaires used as ‘‘gold-standard’’ reference instruments (i.e. the Hospital Anxiety and Depression Scale [HADS] and the Brief Symptom Inventory-18 [BSI 18] (10,11,12). More recently, by studies carried out in Japan (13) , the Netherlands (14) , France (15) ,Turkey (16) , Korea (17) ,Taiwan (18) , Australia (19) , Denmark (20) , Iceland (21), Israel (22) and Ireland (23), the DT’s validity has been confirmed and applied in many countries. Because the DT and PL have never been submitted to a nationwide validation study in Iran, the objective of the current study considering to examination the validity and acceptance of the DT in a large sample of patients of Shiraz with cancer.

## Material and methods

This study involved 38 cancer centers in *AMIR *Oncology hospital in Shiraz. Which was conducted in a 2-day period during an index week at all centers (November 20-27, 2010). Criteria for recruitment included age between ages 18 and 75 years, a primary diagnosis of cancer, a Karnofsky performance status ≥80, a schedule for an outpatient appointment and the ability to provide informed consent. The study was first approved by the ethical committee of the coordinating center. then short, individual, semi-structured clinical interview was conducted by a research psychologist ,who had clinical experience in order to obtain information such as the presence of life-time psychological disorders, the occurrence of stressful events within the last 6 years ago (with the exclusion of events related to cancer) and the current use of psychotropic drugs.

Another source of data (e.g. disease stage, type of therapy, and medical comorbidity) applied in this study was obtained through patients’ medical records with the help of an oncologist who knew the patients. 

Subsequently information as well as patient’s information in medical record was coded in a yes/no format. Finally each patient was asked to complete a booklet containing the following psychological instruments: the DT, the PL, the HADS, and the BSI-18. In the following, each instrument is elaborated in more detail.

**The Distress Thermometer**

The DT is a visual analog tool that asks the respondent to rate his/her level of distress in the past week on a scale from 0 (no distress) to 10 (extreme distress) (8,9). The PL consists of a list of 34 problems grouped into 5 categories (practical problems, family problems, emotional problems, spiritual/religious concerns, and physical problems) and is rated in a yes/no format.

**The Hospital Anxiety and Depression Scale**

HADS is a 14-item, self-report measure of psychological distress divided into 2 subscales: anxiety (7 items) and depression (7 items) (11). For each item, respondents were asked to mark 4 options (rated from 3 to 0; score range, 0-42) which described (closely, exactly) their feeling during the past week. For current study, only the total HADS score Of 15 and 19 were considered to represent clinically significant distress (general caseness) and a more conservative and severe psychiatric conditions (severe caseness), respectively. 

**The Brief Symptom Inventory-18**

BSI-18is an 18-item questionnaire that examines distress through 3 subscales: somatization (6 items), anxiety (6 items), and depression (6 items). Each item is rated on a Likert scale from 0 (‘‘not at all’’) to 4 (‘‘extremely’’), and a total distress score (General Stress Index [GSI]) is obtained by summing all the items. For current study, only GSI was examined; In addition, a T-score ≥63 based on Derogatis was used as indicative of caseness (12). The grade of understandability of the tools also was evaluated by asking patients to answer a single question, ‘‘was this tool easy to understand and to answer?’’ on a 10-point visual analog scale from 0 (‘‘very easy’’) to 10 (‘‘not easy at all’’).

Information about clinical data (e.g. disease stage, type of therapy and medical comorbidity) was obtained from patients’ medical records with the help of an oncologist who knew the patient. 

To understand the possible role of other medical comorbidities on emotional distress, the Charlson comorbidity index (CCI) was used in a modified version according to suggestions made by Watkins et al. Cancer was excluded because it was the primary diagnosis rather than a comorbid condition (24,25). Likewise, dementia was excluded because of the inclusion criteria. For each of the other conditions (rheumatologic disease, chronic pulmonary disease, congestive heart failure, peripheral vascular disease, cerebrovascular disease, diabetes, moderate/severe renal disease, and moderate/severe liver disease), a score was given according to CCI. For the present analysis, the score (i.e. the sum of weights of conditions recorded as being present) was transformed into a 4-level ordinal scale on which the categories 0, 1, 2, and 3 corresponded to index scores of 0, 1 or 2, 3 or 4, and >5, respectively (2).

**Statistical Analysis**

Descriptive analyses along with Pearson correlation tests, chi-square tests, Student Ttests, and analyses of variance were considered as appropriate to examine correlations and differences between groups. Receiver operating characteristic (ROC) analysis was used to determine whether scores on the DT could validly distinguish ‘‘cases’’ and ‘‘noncases’’ as measured by both the HADS and the BSI-18. To do this, the sensitivity and specificity of each score in the range of the DT were calculated and used to determine how well the score distinguished patients who surpassed the HADS and BSI-18 cutoff scores from patients who did not. The ROC curve graphically represents the sensitivity and specificity coefficients that would be generated using each possible cutoff score in the range of DT scores, and the accuracy of the cutoff score was determined by calculating the area under the ROC curve (AUC) (from 1.0 [perfect accuracy] to 0.5 [accuracy no better than chance].

## Results

**Sociodemographic and Clinical Characteristics**

Among 71 patients who were eligible for inclusion, 6 (11%) declined participation, and 7 (11%) were excluded because their psychometric data were incomplete. The final sample consisted of 58 patients men (30%) and 40(70%) women; mean age, 53.4-9.3 years). Patients' Sociodemographic and clinical data were considered.

**Establishment of Distress Thermometer**

The frequency distribution of DT scores is reported in Table 1. The mean (_standard deviation) score on the DT was 4.13 _ 2.92.

**Figure 1:**
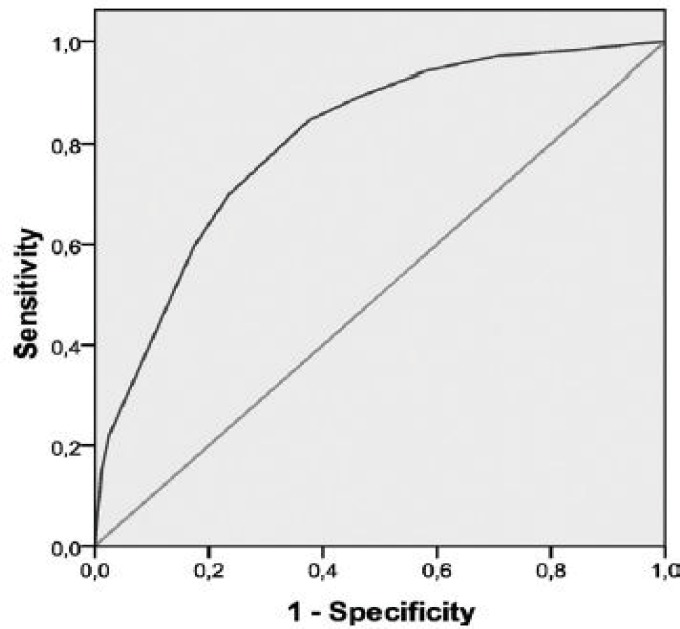
Receiver operating characteristic analysis of caseness on the Hospital Anxiety and depression Scale is illustrated based on cutoff score of 15.

As it is clear in figure 1 In our ROC analysis, a DT cutoff score ≥4 yielded a sensitivity of 0.79 with moderate specificity (0.60) when a HADS cutoff score ≥15 was used as a criterion for general caseness, with an AUC of 0.76 (95% confidence interval [CI], 0.73-0.79) (Fig.1).

**Figure 2:**
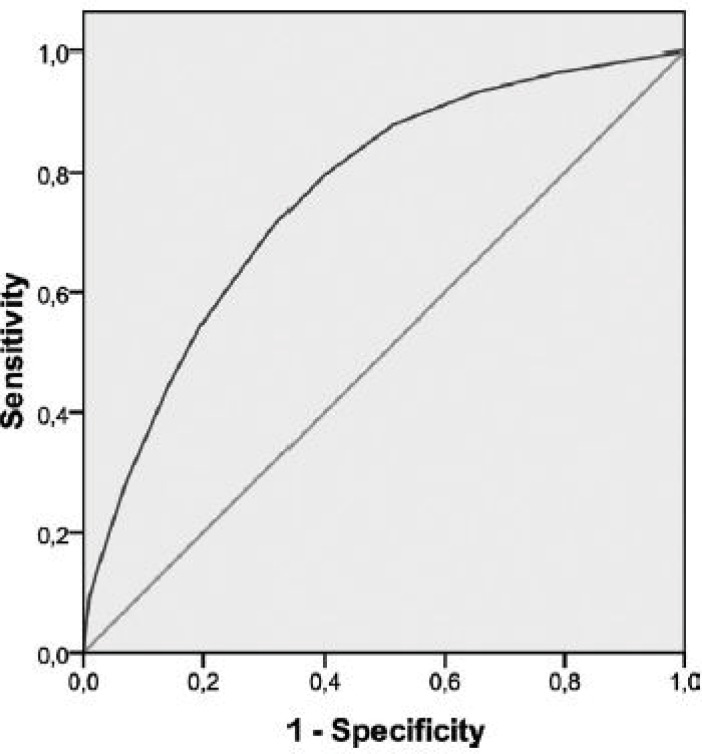
Receiver operating characteristic analysis of caseness on the 18-item Brief Symptom Inventory is illustrated based on a cutoff T-score _63.

The same DT cutoff score ≥4 was associated with a sensitivity of 0.80 and a specificity of 0.61 in identifying cases according to a BSI-18 cutoff T-score ≥63 (AUC, 0.76; 95% CI, 0.73-0.79) (Fig. 2). Therefore, 47% of our patients fulfilled criteria for caseness on the DT.

When a conservative cutoff score of >19 was used on the total HADS as a reference criterion, more severe cases were identified by a DT cutoff score >5, with a sensitivity of 0.70 and a specificity of 0.77 (AUC, 0.81; 95% CI, 0.77-0.83). In this analysis, 33% of patients had distress identified according to the DT. Correspondence of the DT with HADS and BSI-18 scores is shown in Table 2.

**Correlation of Distress Thermometer Cutoff Scores With Clinical and Sociodemographic Variables**

Patients who reported psychological problems in the past and the occurrence of life-events in the last year were more likely to have scores ≥4 on the DT. Correlation of Distress Thermometer Caseness to Problem List Items According to the study by Jacobsen et al. we examined the relation of DT scores with yes/no responses to the list of problems (10). With regard to practical problems, the DTcutoff score was not related to any of the 6 problems listed (0%). considering relations, the DT cutoff score was related significantly to 2 of 4 family problems (50%) (Dealing with partner, P =.001; dealing with children, P =.004). In the area of emotional problems, the DT cutoff score was related significantly to all the problems listed (depression, nervousness, sadness, worry, and loss of interest in usual activities; P =.001). In terms of spirituality, the DT cutoff score was related significantly to spiritual problems and emotional concerns. The DT cutoff score was also associated with 10 of 21 physical problems listed (47%) (problems with appearance, constipation, eating, fatigue, feeling swollen, getting around, indigestion, memory and concentration, pain, and sleep; P =.001).

**Understand Ability and Ease of Using the Tools**

All of the instruments we used for evaluation were quite easy to be understood and answered >80% of patients endorsed this view however A slight but significant preference was demonstrated for the DT (t =2.84; P <.01).

**Table I:** Frequency of Distress Thermometer Scores.

**Scale**	**<DT Score** **Cutoff**	**>DT Score** **Cutoff**	**Chi-Square**	**p-value**
**HADS score**			161.5	0.001
**<Cutoff **	15465(68)	218(32)		
**>Cutoff **	15122(29)	303(71)		
**HADS score **			168.8	0.001
**<Cutoff **	19672(76)	212(24)		
**>Cutoff **	1967(30)	157(70)		
**BSI-18 score**			153.9	0.001
**<Cutoff **	476(67)	235(33)		
**>Cutoff **	111(28)	286(72)		

**Table II:** Correspondence of the Scores on the Distress Thermometer with Cutoff Scores on the Hospital Anxiety and Depression Scale and the Brief Symptom Inventory-1.

**Distress Thermometer Score **	**No. of Patients (%)**
**0**	8(14)
**1**	6(10)
**2**	6(11)
**3**	6(10)
**4**	5(8)
**5**	8(14)
**6**	5(7)
**7**	6(11)
**8**	5(9)
**9**	1(2)
**10**	2(4)

## Discussion

To our knowledge, this is the first wide study done in Shiraz examining the validity of the DT as a screening tool for distress in a large sample of patients with cancer. Like other national evaluations, the DT is identified as a valid tool for detecting distress in Iranian cancer patients compared with standard measures such as HADS and BSI-18. The finding of the present study demonstrated that a cutoff score of ≥4 on the DT maximized sensitivity and specificity for general psychosocial morbidity, with an AUC on both the HADS and BSI-18 that indicated acceptable accuracy of the DT. Among our patients, 47% had possible distress on the DT, and they had a tendency to overestimate caseness as measured by the HADS and BSI-18 (33% and 38%, respectively). Using more conservative cutoff score for more severe caseness (i.e. a HADS score _19), a score _5 on the DT maximized sensitivity and specificity with an AUC of 0.80, the obtained results indicated significant distress in 33% of patients.

When distress on the DT was rated as mild, moderate, or severe, the proportion of patients with severe distress was greater than what was reported in the study carried out by Mitchell et al. in the United Kingdom (6). The differences in DT caseness and cutoff scores between studies are not easy to interpret among current studies. Because many variables can influence levels of distress (e.g. the study setting, possible clinical factors), further studies on more homogeneous populations are necessary.

In our investigation, distress was not related to age, education, marital status, stage of cancer, or type of intervention; it is also interesting to note that distress was not related to medical comorbidity. The latter is an important finding, because no other study tried to examine the possible role of other concomitant diseases in influencing scores on the DT in patients with cancer. Our study showed that distress was greater in women than in men and was associated with previous psychological disorders and the occurrence of stressful life events, other than cancer, in the year preceding the diagnosis of cancer. Cancer-related problems, including relational, emotional, spiritual, and physical problems, which emerged by administering the PL, were also more markedly evident among patients who had distress identified by the DT than among patients without such distress.

These findings are in line with data indicating that the presence of physical symptoms can increase the risk of distress (10). Likewise; the impact of cancer on interpersonal and individual-spiritual dimensions was also examined in several other studies, which confirmed the association of cancer with spiritual distress, emotional distress, and maladjustment to cancer (28-30). On the other hand, distress may overlap with problems in the domain of psychological symptoms on the PL, such as depression, nervousness, sadness, and concern about health, as indicated by our current results.

Regarding the intelligibility and clarity of the tools, our patients reported that the instruments were understandable and easy to complete. The recognition of distress and the proper referral rate of cancer patients in clinical follow up (31). With respect to this finding, an educational program on routine use of the DT identification and the referral of patients with distress (from 7% to 23%) by psycho-oncology services (32). These results confirmed what has been reported from some other countries. Indicating an increase in referrals for distress problems after introduction of the DT in clinical several areas of the country. In addition, to our knowledge, this study is the first in the DT literature to examine the possible role of medical comorbidity and the acceptability of the tools by patients with cancer.

**Limitations of the Study**

The present study is compromised by some limitations, for example, the sample. The sample consisted of cancer outpatients with a good performance status, although half of them were in a metastatic disease phase.

However, the only available Iranian study relative to using the DT versus a psychiatric diagnosis based on the World Health Organization-Composite Interview for the International Classification of Diseases, 10th edition, confirmed the data presented here (37). Third, the cancer site was represented mostly by breast and gastrointestinal cancer, and this did not allow us to understand, in amore comprehensive way, the possible differences in DT scores using cancer sites. the present finding is in contrast with Zabora et al. conducted a large study involving 9000 patients with cancer and reported higher distress rates among patients with lung cancer (43%) and lower rates among patients with gynecologic cancer (30%), because the percentages of women and patients in a metastatic stage of illness were high in our study, this may have had an impact on our results (26). Further study taking into account sex, patient age, and disease stage are necessary.

Even, if the methodology of other research was followed, it could be improved, as recently suggested by Brennan et al. (38). Fifth, more specific data on previous and current psychiatric history (e.g. specific diagnosis of substance abuse disorders, bipolar disorders, schizophrenia, and type of psychotropic medications) and categories of stressful events will be important to identify the true distress level in patients with cancer. Finally, we are aware that traditional criteria for a screening tool (e.g. high specificity, high sensitivity, ‘‘do no harm’’ to patients, ease of use, and effectiveness) need to be fulfilled before daily application in the clinical setting. These aspects also should be applied to the DT. In addition, both referral and treatment algorithms need to be developed. The role of the DT in cancer patients of Shiraz with poorer performance status and in the context of palliative care should be examined in further studies. Second, although we followed the existing literature in using psychometric questionnaires as reference criterion for caseness, more information is needed regarding the accuracy of the DT with respect to a standard psychiatric interview. 

## Conclusion

In conclusion, in this study, we confirmed that a brief screening tool like the DT is a simple and effective screening instrument for detecting distress in Iranian patients with cancer. The instrument is easy to understand and thereby promises high compliance among both patients and clinicians. Furthermore, the single item DT compares favorably with longer measures that are used to screen for distress and that, when combined with the PL, favors the identification of cancer-related problems. There is a need for studies in Iranian examining in depth the outcome of identifying distress in cancer patients in terms of both referral rates and, especially, treatments Patients indicated that the DT was easier to fill out and to understand than the HADS, but not the BSI-18. The DT was identified as a simple and effective screening instrument for detecting distress in Iranian cancer patients.
